# Supplementary material for: Defining interstitial lung disease related to idiopathic inflammatory myopathies: a systematic review protocol of the Myositis Clinical Trial Consortium (MCTC)
Source: Syst Rev. 2026 Jun 26;15:252. doi: 10.1186/s13643-026-03230-w (PMC13573363; doi:10.1186/s13643-026-03230-w)
Supplement: Supplementary file 1 [file 13643_2026_3230_MOESM1_ESM.docx]

**Defining Interstitial Lung Disease Related to Idiopathic Inflammatory Myopathies: A Systematic Review Protocol of the Myositis Clinical Trial Consortium (MCTC)**

Konstantinos Triantafyllias^1,2^, Ramiro Gomez^3^, Jisna Paul*^4^, Simone Appenzeller^5^, Elena Bartoloni^6^, Alejandro Benitez^7^, Daniel Brito de Araujo^8^, Solciris A. Dominguez^9^, Komal Ejaz^10^, Antonis Fanouriakis^11^, Kristin Houghton^12^, Marc Janssen^13^, Chandana Keshavamurthy^14^, Marina Khoury^15^, Shaye Kivity^16,17^, Océane Landon-Cardinal^18^, Ignacio Lopez^19^, Nikolaos Marketos^20,21^, Edoardo Marrani^22^, Jan Willem Marsden^13^, Clio Mavragani^21^, Pintip Ngamjanyaporn^23^, Apostolos Perelas^24^, Victor Pimentel-Quiroz^25,26^, Vasilios Tzilas^27^, Juan David Urriago Gil^28^, Susan Vehar^29^, Gwen Wilson^30^, Erin Wilfong^31^, Charalampia Papadopoulou^32^, Rohit Aggarwal^33^, Robert Hallowell^34^, Vivek Nagaraja^35^, Faye Pais**^36^, Elena K. Joerns**^37^, on behalf of the Myositis Clinical Trials Consortium [IIM-ILD definition group].

^*^Corresponding author

^**^Shared senior authors

1. Department of Rheumatology, Rhineland-Palatinate Rheumatology Center, Kaiser-Wilhelm-Straße 9-11, 55543 Bad Kreuznach, Germany.
2. Division of Rheumatology and Clinical Immunology, Department of Internal Medicine I, Johannes Gutenberg University Medical Center, Langenbeckstraße 1, 55131 Mainz, Germany.
3. Rheumatology Department, Austral University Hospital, Buenos Aires, Argentina.
4. Rheumatology & Immunology, Ohio State University Wexner Medical Center, Columbus, Ohio, USA.
5. Department of Orthopedics, Rheumatology and Traumatology, School of Medical Science, University of Campinas, Rua Tessália Vieira de Camargo 126, Cidade Universitária, 13083-887 Campinas, São Paulo, Brazil.
6. Rheumatology Unit, Department of Medicine and Surgery, University of Perugia, Piazzale Giorgio Menghini 1, 06129 Perugia, Italy.
7. Rheumatology Service, Posadas Hospital Nacional, Buenos Aires, Argentina.
8. Department of Internal Medicine, Faculty of Medicine, Federal University of Pelotas, Avenida Duque de Caxias 250, Fragata, 96030-001 Pelotas, RS, Brazil.
9. Division of Rheumatology and Clinical Immunology, University of Pittsburgh School of Medicine, Pittsburgh, PA, USA.
10. Department of Rheumatology, Piedmont Healthcare, 1270 Prince Avenue, Athens, GA, 30606.
11. Rheumatology-Clinical Immunology Unit, Attikon General University Hospital, School of Medicine, National and Kapodistrian University of Athens, Greece.
12. Division of Rheumatology, Department of Pediatrics, BC Children’s Hospital and University of British Columbia, 4480 Oak Street, Vancouver, BC V6H 3V4, Canada.
13. Division of Pediatric Rheumatology and Immunology, Wilhelmina Children’s hospital, UMC Utrecht, Utrecht, The Netherlands Department of Rheumatology
14. Department of Rheumatology, Banner University Medical Center, University of Arizona, Phenix, AZ, USA
15. Alfredo Lanari Medical Investigations Institute, Autonomous City of Buenos Aires, Argentina.
16. Rheumatology Unit, Meir Medical Center, 59 Tchernichovsky Street, Kfar Saba 4428164, Israel.
17. Faculty of Medical and Health Sciences, Tel Aviv University, P.O. Box 39040, Ramat Aviv, Tel Aviv 6997801, Israel.
18. Division of Rheumatology, Department of Medicine, Centre Hospitalier de l’Université de Montréal, 900 rue Saint-Denis, Pavillon R, Montréal, QC H2X 0A9, Canada.
19. Rheumatology Department, Sanatorio Güemes, Autonomous City of Buenos Aires, Argentina.
20. Rheumatology Outpatient Department, Henry Dunant Hospital Centre,107 Mesogeion Avenue, GR-115 26 Athens, Greece.
21. Department of Physiology, School of Medicine, National and Kapodistrian University of Athens, 75 Mikras Asias Street, GR-115 27 Goudi, Athens, Greece.
22. Pediatric Rheumatology Unit, Meyer Children’s Hospital IRCCS, Viale Gaetano Pieraccini 24, 50139 Florence, Italy.
23. Division of Allergy, Immunology, and Rheumatology, Department of Medicine, Faculty of Medicine, Ramathibodi Hospital, Mahidol University, 270 Rama VI Road, Thung Phaya Thai, Ratchathewi, Bangkok 10400, Thailand.
24. Division of Pulmonary Disease and Critical Care Medicine, Virginia Commonwealth University, Richmond, VA 23298, USA.
25. Grupo Peruano de Estudio de Enfermedades Autoinmunes Sistémicas, Universidad Científica del Sur, Lima, Peru.
26. Department of Rheumatology, Hospital Nacional Guillermo Almenara Irigoyen, Essalud, Lima, Peru
27. 2nd Pulmonary Medicine Department, School of Medicine, Attikon General University Hospital, National and Kapodistrian University of Athens, 1 Rimini Street, GR-124 62 Chaidari, Athens, Greece.
28. Rheumatology Service, San Ignacio University Hospital, Bogota, Colombia.
29. Division of Pulmonary, Critical Care and Sleep Medicine, University of Miami Miller School of Medicine, Miami, Florida, USA.
30. Mayo Clinic Libraries, Mayo Clinic, Rochester, MN, USA.
31. Division of Rheumatology and Immunology and Division of Allergy, Pulmonary, and Critical Care Medicine, Department of Medicine, Vanderbilt University Medical Center, Nashville, TN 37232, USA.
32. Department of Rheumatology, Great Ormond Street Hospital for Children NHS Foundation Trust and UCL GOS Institute of Child Health, London, UK.
33. Division of Rheumatology and Clinical Immunology, Department of Internal Medicine, University of Pittsburgh, Pittsburgh, PA, UA.
34. Division of Pulmonary and Critical Care, Department of Medicine, Massachusetts General Hospital, Harvard Medical School, Boston, Massachusetts, USA.
35. Division of Rheumatology, Department of Internal Medicine, Mayo Clinic, Scottsdale, Arizona, USA.
36. Division of Pulmonary, Critical Care, and Sleep Medicine, Department of Medicine, University of Florida College of Medicine, 1600 SW Archer Road, Gainesville, FL 32608, USA.
37. Division of Rheumatology, Department of Internal Medicine, Mayo Clinic, Rochester, Minnesota 55905, USA.

**Corresponding author:**

Jisna Paul, Rheumatology & Immunology, Ohio State University Wexner Medical Center, Columbus, Ohio, USA

**Email:** [Jisna.Paul@osumc.edu](mailto:Jisna.Paul@osumc.edu)

**Abstract**

**Background**

Idiopathic inflammatory myopathies (IIM) are systemic autoimmune diseases that include dermatomyositis, polymyositis, antisynthetase syndrome, and immune-mediated necrotizing myopathy in both adult and juvenile populations. Interstitial lung disease (ILD) is a common and serious complication of IIM, with a highly variable clinical course ranging from indolent disease to rapidly progressive respiratory failure, often influenced by underlying myositis-specific autoantibody profiles. Despite the substantial morbidity and mortality associated with IIM-ILD in both adults and children, high-quality evidence to guide clinical management remains limited. A major barrier to progress in this field is the lack of standardized disease nomenclature and uniform diagnostic or classification criteria, which hampers cohort development, limits comparability across studies, and restricts generalizability of findings. To address this gap, we will conduct a systematic review to inform a consensus-based disease definition for adult and juvenile IIM-ILD, with detailed methodology outlined in this protocol. This review will not evaluate prognosis, treatment, or clinical outcomes, but instead focus exclusively on how IIM-ILD is defined and operationalized across studies.

**Methods**
Following PRISMA 2020 statement and Cochrane methodology, we will search PubMed, EMBASE, Scopus, Cochrane Systematic Reviews, and Cochrane Central from inception to June 30, 2025, using a ‘‘Peer Review of Electronic Search Strategies (PRESS)’’ - validated strategy. Eligible studies will include randomized controlled trials (RCTs), cohort and case-control studies, systematic reviews, meta-analyses, guidelines, and consensus statements explicitly reporting IIM-ILD definitions in human participants of all ages. Study selection will be performed in Covidence using predefined criteria. Data extraction will capture case definitions, classification criteria, diagnostic methods, ILD patterns, autoantibody profiles, and study characteristics. Quantitative synthesis will summarize the frequency and components of definitions where possible; otherwise, a structured narrative synthesis will be performed.

**Discussion**
Our systematic review protocol is the first comprehensive attempt at synthesizing the highly heterogenous disease nomenclature currently employed in adult and juvenile IIM-ILD literature. Findings will map areas of agreement and inconsistency in existing definitions, forming an evidence base for subsequent consensus processes, such as Delphi, to establish standardized nomenclature. Harmonized definitions are expected to improve research reproducibility, facilitate multicentric collaboration, and improve patient stratification in clinical practice and trials.

**Systematic review registration:** PROSPERO ID: 1129967

**Keywords:** idiopathic inflammatory myopathies, interstitial lung disease, myositis-specific autoantibodies, myositis-associated autoantibodies, definition, classification, protocol, systematic review

**Background**

Idiopathic inflammatory myopathies (IIM) are a heterogeneous group of systemic autoimmune disorders that include, but are not limited to, dermatomyositis (DM), polymyositis, antisynthetase syndrome (ASyS), and immune-mediated necrotizing myopathy (IMNM) (adult and juvenile forms) (1). Although myositis is traditionally defined by muscle inflammation and weakness, this terminology is misleading because many patients exhibit extramuscular manifestations - such as skin, lung, and joint involvement - with interstitial lung disease (ILD) in IIM potentially occurring before, concurrently with, after, or even independently of classic muscle or skin symptoms. Of these, ILD is one of the most serious and potentially life-threatening complications associated with IIM in both adults and children (2, 3).

IIM-associated ILD (IIM-ILD) can present in various forms, namely acutely, subacutely, or insidiously, and significantly contribute to disease morbidity and mortality. Early recognition and management are crucial due to the variability in presentation and the risk of rapid progression, especially in subtypes like anti-MDA5 (anti-Melanocyte Differentiation-Associated protein 5) DM and ASyS (4). This is particularly true for individuals who test positive for some specific myositis-related autoantibodies (MSA) as well (5, 6). Early identification of ILD in these patients is essential, as prompt initiation of immunosuppressive therapy can improve outcomes and reduce morbidity (2).

The spectrum of idiopathic inflammatory myopathy–associated interstitial lung disease and related entities is summarized in Figure 1.

Figure 1. IIM subsets, ILD patterns, relationship to autoantibodies, overlap with IPAF and other SARD-ILD subtypes. (BioRender was used for figure preparation). For a glossary of key terms used in this manuscript also refer to Supplementary file 2

*(PM, polymyositis; DM, dermatomyositis; AS, antisynthetase syndrome; IBM, inclusion body myositis; IIM, idiopathic inflammatory myopathy; IIM-ILD, idiopathic inflammatory myopathy–associated interstitial lung disease; IPAF, interstitial pneumonia with autoimmune features; ILD, interstitial lung disease; UIP, usual interstitial pneumonia; NSIP, non-specific interstitial pneumonia; OP, organizing pneumonia; LIP, lymphocytic interstitial pneumonia; anti-Jo-1, histidyl–transfer RNA (tRNA) synthetase; anti-PL-7, threonyl-tRNA synthetase; anti-PL-12, alanyl-tRNA synthetase; anti-EJ, glycyl-tRNA synthetase; anti-OJ, isoleucyl-tRNA synthetase; anti-KS, asparaginyl-tRNA synthetase; anti-Zo, phenylalanyl-tRNA synthetase; anti-Ha, tyrosyl-tRNA synthetase; anti-Ly, lysyl-tRNA synthetase; anti-VR, valyl-tRNA synthetase; anti-Mi-2, nucleosome remodeling–deacetylase complex; anti-MDA5, melanoma differentiation–associated protein 5; anti-TIF1-γ, transcription intermediary factor 1-gamma; anti-NXP2, nuclear matrix protein 2; anti-SAE, small ubiquitin-like modifier–activating enzyme; anti-SRP, signal recognition particle; anti-HMGCR, 3-hydroxy-3-methylglutaryl–coenzyme A reductase; anti-PM-Scl, polymyositis–scleroderma overlap; anti-Ku, Ku heterodimer; anti-U1RNP, U1 ribonucleoprotein; anti-U3 RNP (fibrillarin), U3 ribonucleoprotein; anti-Sm, Smith antigen; anti-RNA polymerase, RNA polymerase; anti-Ro52 (TRIM21), tripartite motif–containing 21; MAA, myositis-associated antibodies; SARD, systemic autoimmune rheumatic disease; SLE, systemic lupus erythematosus; RA, rheumatoid arthritis)*

The latest guidelines from the American College of Rheumatology (ACR) and the American College of Chest Physicians (CHEST), and the SHARE (Single Hub and Access point for paediatric Rheumatology in Europe) initiative for children (7), stress the importance of screening, monitoring, and treating ILD in patients with systemic autoimmune rheumatic diseases (SARDs), including IIM (8, 9). However, these guidelines also acknowledge the lack of standardized screening protocols and specific diagnostic criteria for IIM-ILD. While IIM are recognized as a subset of SARDs at high risk for developing ILD, the guidelines do not offer a consensus definition for IIM-ILD (8).

The lack of consensus definition for IIM-ILD impedes epidemiological studies of its prevalence and incidence, due to inconsistent application of the definitions in literature. Additionally, observational studies of treatments for IIM-ILD are difficult to interpret with heterogeneous IIM-ILD definitions across studies. Therefore, development of consensus definition would significantly improve the understanding of IIM-ILD epidemiology and behavior.

Importantly, differentiating IIM-ILD from interstitial pneumonia with autoimmune features (IPAF) can be challenging, due to overlapping clinical, radiologic, and serologic findings, particularly MSA positivity (10). The inclusion of these antibodies in the serologic criteria for IPAF has complicated the differentiation between IPAF and IIM-ILD. Research suggests that patients with IPAF who test positive for MSA are more effectively managed within the IIM-ILD framework. This is because their prognosis, treatment responses, and monitoring requirements align with IIM-ILD and differ significantly from those of other IPAF phenotypes (11).

IIM-ILD warrants its own definitional framework, rather than being considered within SARD-ILD or IPAF constructs. IIM-ILD has specific subtypes with unique clinical behaviors. As such, survival in IIM-ILD may be affected by the type of autoantibody, with MSA presence portending improved prognosis as compared to MAA (11), and with most notable antibody being anti-melanoma differentiation-associated gene 5 (MDA-5) antibody, which is associated with amyopathic dermatomyositis and rapidly progressive ILD (3). This unique association of autoantibodies with clinical trajectory in IIM-ILD has significant implications for homogeneous trial recruitment and appropriate endpoint selection, reinforcing the necessity of homogeneous application of IIM-ILD definition in studies and cohorts.

Addressing the diagnostic and classification gaps within IIM-ILD has been a focus of collaborative international efforts. The Myositis Clinical Trials Consortium (MCTC) is a multidisciplinary alliance of clinicians, researchers, patient groups, and industry partners, dedicated to improving clinical trial design and related research in IIM (12). By bringing together a diverse network of investigators from different regions, disciplines, and career stages, MCTC aims to accelerate the development of evidence-based treatments and improve clinical trial readiness in IIM. Its Steering Committee, Core Group, and Pediatric Subcommittee comprise established experts, early- and mid-career researchers worldwide, working in coordination with other myositis organizations to complement existing initiatives and focus on the practical aspects of trial design and execution.

The absence of a universally accepted nomenclature for adult and juvenile IIM-ILD significantly impedes research progress and clinical trial development and highlights the critical need for concerted efforts focused on establishing a consensus-based standard. The IIM-ILD working group (WG) of the MCTC is a lung-centered initiative focused on addressing unique challenges facing adults and children with IIM. As such the IIM-ILD WG has leveraged the MCTC’s vast global expertise to address the challenge and has proposed a framework to achieve a uniform and universally accepted IIM-ILD nomenclature. This protocol outlines the first step toward that goal by defining the approach for a systematic literature review (SLR) that will examine existing definitions, diagnostic criteria, and classification frameworks for IIM-ILD in adults and children. For the purposes of this review, IIM-ILD will be defined operationally as ILD occurring in the setting of IIM, as characterized by the source study. The review will distinguish core IIM-ILD from related but conceptually adjacent entities, including overlap CTD-ILD, IPAF-like presentations, and pulmonary disease in patients with IIM primarily attributed to non-autoimmune causes such as drug toxicity or infection, where such distinctions are explicitly reported.

**Methods**

*Conducting guidelines*

This protocol will adhere to the Preferred Reporting Items for systematic review and meta-analysis protocols (PRISMA-P) 2015 statement which are fully detailed in the supplementary checklist and follow methodological principles outlined in the Cochrane Handbook for Systematic Reviews of Interventions (13). The protocol is registered in PROSPERO (ID: 1129967). Anticipated protocol amendments will be prospectively documented, and transparently reported in the final manuscript. The SLR will adhere to the PRISMA 2020 statement (14), and will be conducted according to a predefined timeline: literature search and study screening will be completed within the first 3 months, data extraction and risk of bias assessment within months 4–5, and data synthesis and manuscript preparation within months 6–7. Extracted data and analytic materials will be provided as supplementary files and archived in a long-term open-access repository (e.g., OSF or Zenodo) to ensure transparency, reproducibility, and data preservation.

*Eligibility criteria and benchmarking references*

This review will include studies involving individuals of all ages from the general population who are identified as having IIM-associated interstitial lung disease (IIM-ILD). Guidelines, consensus statements, and classification proposals will be included, as these sources frequently provide explicit disease definitions and diagnostic frameworks that are applied in both clinical practice and research settings. Because the primary objective of this review is to examine how IIM-ILD is defined and classified, rather than to evaluate therapeutic outcomes, such documents are critical for understanding the operational criteria used to identify IIM-ILD across studies and clinical trials.

The primary outcome is the definition of adult and juvenile IIM-ILD, and eligible studies must focus on definition, classification, or diagnostic criteria. Publications will be sourced from PubMed (1966–June 30, 2025), EMBASE (1947–June 30, 2025), SCOPUS (inception–June 30, 2025), Cochrane Systematic Reviews (inception–June 30, 2025), and Cochrane Central (inception–June 30, 2025). Included article types will be randomized controlled trials (RCTs), cohort studies, and case-control studies, as well as meta-analyses, systematic reviews, guidelines, consensus statements, and classification proposals, published in English, and involving human participants. June 30, 2025 cut-off date is chosen as it was the date of the first meeting of the author group and had no relation to the Delphi timeline. Given that the majority of authors conduct research and publish on the topic of this systematic review (IIM-ILD), this cut-off date is designed to prevent the bias in definition arising due to involvement in this project.

Non-peer-reviewed literature, case reports, small case series (adult) with fewer than 10 patients, pediatric studies with fewer than 5 participants, narrative reviews, editorials, expert opinions, and consensus papers without primary data will be excluded, due to their limited ability to provide standardized or generalizable definitions. Moreover, no grey literature, such as thesis, dissertations, technical reports, conference abstracts, or other non-peer-reviewed sources, will be included. Non-English publications will also be excluded to ensure feasibility and consistency in data extraction across all review team members, particularly in cases requiring resolution of disagreements, and to minimize the risk of misclassification when evaluating definitions of IIM-ILD. No restrictions will be placed on the diagnostic or classification criteria used to define IIM (e.g., Bohan and Peter, ENMC, or ACR/EULAR criteria); rather, these will be systematically extracted and compared to evaluate their influence on IIM-ILD definitions across studies. Studies including overlap syndromes (e.g., IIM with systemic sclerosis or other connective tissue diseases) will be included if IIM-ILD is defined as a distinct subgroup. Studies in which pulmonary involvement is attributed primarily to alternative causes (e.g., infection or drug-induced lung injury) rather than ILD related to IIM will not contribute to the core definitional synthesis, although such attribution will be recorded when reported. Adult and juvenile populations will be analyzed separately to account for differences in disease phenotype, diagnostic approaches, and reporting practices.

Table 1 demonstrates benchmarking articles for the inclusion criteria.

**Table 1.** Benchmarking articles (15-21).

Correia BP, Campanilho-Marques R, Dourado E, Silva M, Silva A, Costa F, et al. Myositis-associated interstitial lung disease: the experience of a tertiary center. J Clin Med. 2024;13(20):6085 (15)

Tian J, Ha YJ, Lee JS, Lee EY, Goldin JG, Kim GJ. Application of a growth-rate model to enhance subgroup identification in heterogeneous clinical courses of the idiopathic inflammatory myopathy-associated interstitial lung disease and its prognostic implications. Int J Rheum Dis. 2025;28(6):e70320 (16)

Zhang W, Huang G, Zheng S, Lin J, Hu S, Zhuang J, et al. Risk prediction modelling in idiopathic inflammatory myositis-associated interstitial lung disease based on seven factors including serum KL-6 and lung ultrasound B-lines. Clin Exp Rheumatol. 2025;43(2):260–8 (17)

Wang H, Wang Y, Sun D, Yu S, Du X, Ye Q. Progressive pulmonary fibrosis in myositis-specific antibody-positive interstitial pneumonia: a retrospective cohort study. Front Med. 2024;10:1325082 (18)

Selva-O'Callaghan A, Labrador-Horrillo M, Muñoz-Gall X, Martínez-Gomez X, Majó-Masferrer J, Solans-Laque R, et al. Polymyositis/dermatomyositis-associated lung disease: analysis of a series of 81 patients. Lupus. 2005;14(7):534–42 (19)

Aggarwal R, McBurney C, Schneider F, Yousem SA, Gibson KF, Lindell K, et al. Myositis-associated usual interstitial pneumonia has a better survival than idiopathic pulmonary fibrosis. Rheumatology (Oxford). 2017;56(3):384–9 (20)

Hannah JR, Lawrence A, Martinovic J, Naqvi M, Chua F, Kouranos V, et al. Antibody predictors of mortality and lung function trends in myositis spectrum interstitial lung disease. Rheumatology (Oxford). 2024;63(11):3080–90 (21)

*Information Sources and Search Strategy*

The aforementioned core databases (PubMed, EMBASE, Scopus, Cochrane Systematic Reviews, and Cochrane Central) will be searched using a strategy based on the PICO framework.

Our search strategy will include all relevant keywords and synonyms related to IIM, relevant autoantibodies, and ILD. We will ensure inclusion of juvenile IIM by including both pediatric and adult studies. Our age-inclusive (eliminating any age criteria for studies) search strategy was confirmed by a medical librarian and pediatric reviewers to capture key juvenile IIM literature using core disease terms alone, without using specific juvenile IIM and juvenile DM terminology. Also, adding juvenile-specific terms increased non-relevant results without improving recall. We will include the full search strategy in the supplement. Two study authors will ensure the search strategy meets every item on the Peer Review of Electronic Search Strategies (PRESS) checklist (22) before conducting the review. If more than 6 months have elapsed by the time of manuscript submission, we will re-run the search. Search terms are listed in Table 2 below.

**Table 2.** Complete Search Strategy for Pubmed.

| 1 | "Myositis" OR "myositides*" OR "inflammatory myopath*" OR "inflammatory muscle disease*" OR "autoimmune myopath*" OR "anti tRNA synthetase syndrome" OR "anti Jo1 syndrome" OR "Jo1 antisynthetase syndrome" OR "antisynthetase syndrome" OR "anti synthetase syndrome" OR Dermatomyositis OR Polymyositis OR "immune mediated necrotizing myopath*" OR "necrotizing autoimmune myopath*" OR "tRNA Synthetase" OR "Anti Jo 1" OR "Anti Jo1" OR "Anti PL7" OR "Anti PL 7" OR "Anti PL 12" OR "Anti PL12" OR "Anti MDA5" OR "Anti MDA 5" OR "anti SRP" OR "Anti SAE" OR "CADM140" OR "CADM 140" OR "Anti HMGCR" OR "Anti OJ" OR "Anti EJ" OR "Anti KS" OR "Anti Ku" OR "Anti Zo" OR "Anti Ha" OR "Anti NXP2" OR "Anti NXP 2" OR "Anti TIF1gamma" OR "Anti TIF1 gamma" OR "Anti SSA" OR "Anti Ro52" OR "Anti Ro 52" OR "Anti Pm/Scl" OR "Anti Pm/Scl100" OR "Anti Pm/Scl 100" OR "Anti Pm/Scl75" OR "Anti Pm/Scl 75" OR "Anti YRS" OR "Anti p155/140" OR "Anti U1 RNP" OR "Anti FHL1" OR "Anti FHL 1" OR "Anti CN1A" OR "Anti CN 1A" OR "Anti RuvBL1" OR "Anti RuvBL 1" OR "Anti RuvBL2" OR "Anti RuvBL 2" OR "Myositis"[Mesh] |
| --- | --- |
| 2 | "interstitial lung disease*"[Title/Abstract] OR "ILD"[Title/Abstract] OR "diffuse parenchymal lung disease*"[Title/Abstract] OR "interstitial pneumonia*"[Title/Abstract] OR "interstitial pneumonitis*"[Title/Abstract] OR "pulmonary fibrosis*"[Title/Abstract] OR "diffuse alveolar damage"[Title/Abstract] OR "IPAF"[Title/Abstract] OR "organizing pneumonia*"[Title/Abstract] OR "Lung Diseases, Interstitial"[Mesh] OR "Pulmonary Fibrosis"[Mesh] |
| 3 | 1 AND 2 |
| 4 | 3 AND (english[Filter]) |

*Jo-1 – anti-histidyl tRNA synthetase antibody; PL-7 – anti-threonyl tRNA synthetase antibody; PL-12 – anti-alanyl tRNA synthetase antibody; OJ – anti-isoleucyl tRNA synthetase antibody; EJ – anti-glycyl tRNA synthetase antibody; KS – anti-asparaginyl tRNA synthetase antibody; Zo – anti-phenylalanyl tRNA synthetase antibody; Ha / YRS – anti-tyrosyl tRNA synthetase antibody; MDA5 / CADM-140 – anti-melanoma differentiation–associated gene 5 antibody; SRP – anti-signal recognition particle antibody; SAE – anti-small ubiquitin-like modifier–activating enzyme antibody; HMGCR – anti-3-hydroxy-3-methylglutaryl–coenzyme A reductase antibody; Ku – anti-Ku DNA repair protein antibody; NXP2 – anti-nuclear matrix protein 2 antibody; TIF1γ – anti-transcription intermediary factor 1 gamma antibody; SSA – anti-Sjogren’s syndrome antigen A antibody; Ro52 – anti-52 kDa Ro/SSA antibody; Pm/Scl – anti-Pm/Scl complex antibody; Pm/Scl-100 – anti-Pm/Scl 100 kDa subunit antibody; Pm/Scl-75 – anti-Pm/Scl 75 kDa subunit antibody; p155/140 – anti-TIF1γ/TIF1α-β antibody; U1 RNP – anti-U1 ribonucleoprotein antibody; FHL1 – anti-four-and-a-half LIM domains protein 1 antibody; CN1A – anti-cytosolic 5′-nucleotidase 1A antibody; RuvBL1 / RuvBL2 – anti-RuvB-like protein 1/2 antibodies.* *MeSH – Medical Subject Headings for inflammatory muscle diseases.* *IPAF – Interstitial Pneumonia with Autoimmune Features.*

*Study Records and Selection Process*

Database records will be exported to an EndNote library (Clarivate) before uploading to Covidence (23), which automatically deduplicates records. Study selection will be completed in Covidence. All study selection processes will be independently performed by two project members using the inclusion/exclusion criteria. A calibration exercise will be conducted prior to title/abstract using random sample of at least 35 records. Inter-rater agreement will be assessed using Cohen’s κ (threshold ≥0.70), with additional training and subsequent assessment of Cohen's k performed as needed until reliability reaches 0.70 or greater. Following successful completion of the inter-rater reliability test and modifications to the study selection form, all titles/abstracts will be screened by two independent reviewers. Any conflicts will be resolved by a third adjudicator.

A small number of group members will develop a set of questions for screening full-text articles.

Before formal full-text screening begins, a second calibration exercise will be conducted on a random sample of at least 8 full-text articles using Cohen's kappa test. Full-text reviews will follow the same principle as abstract reviews (screening by two independent reviewers and conflict resolvement by a third adjudicator). During the full-text screening process, the articles will be independently reviewed by two team members who will also track reasons for exclusion. Any disagreements on exclusion will be resolved by an additional independent adjudicator.

*Data Collection Process*

Data extraction will be completed by pairs of independent reviewers. A list of potential data points to extract from included full-text articles will be created. A Data Item Team will be assembled, which will narrow the data items proposed and validate the final data items to be extracted using the benchmarking articles. A separate Covidence Data Extraction Management team will be created, and will develop a standardized form for data extraction, as well as a detailed instruction manual for training of reviewers. All reviewers will initially perform data extraction on three to five articles; inter-rater reliability will be assessed, and disagreements between reviewers will be resolved through discussion.

Items on which there is disagreement will be discussed to determine if the reason for disagreement is a training issue or if the data extraction form requires modification. Once the data extraction form is finalized and formal data extraction commences, disagreements will continue to be resolved by an independent adjudicator. In the event of uncertainty about missing or unclear study data, we will contact the study authors to clarify.

*Key Terminology in IIM-ILD*

To clarify the scope of this review, it is important to distinguish between related concepts frequently used in the IIM-ILD literature. In this context, a definition refers to the criteria used to label pulmonary involvement in patients with IIMs; diagnostic criteria encompass the clinical signs, serological markers, imaging findings, and histopathological features used to confirm ILD in an individual patient; and classification criteria are standardized frameworks designed to define patient cohorts consistently for research or clinical trials, ensuring uniformity in case selection across studies.

*Data Items*

For each eligible study, we will extract article and bibliographic information (including author, year of publication, and participating centers) and details on study design, including whether it was an RCT, cohort, case-control, cross-sectional, or longitudinal study. We will also record the sample size and demographic characteristics of study participants such as sex, age, and race. The calendar year(s) of the study will be documented, along with the definition used to determine the study population and the myositis classification criteria applied, myositis subtypes, clinical and serological features, and diagnostic methods employed.

Information on ILD will include the definition applied, diagnostic methods employed, and the ILD pattern reported. To ensure meaningful data comparison and interpretation, only studies reporting High Resolution Computer Tomography (HRCT)-based ILD definition will be included. Should patients with IPAF be included, their frequency will be reported. We will also extract average pulmonary function test (PFT) values and seropositivity rates of different autoantibodies. The presence or absence of a formal IIM-ILD definition within the paper will be recorded. Formal definition will be considered when the authors present an explicit statement of how they defined the IIM-ILD population within their study. A thorough description of the data items to be extracted is presented in Table 3. Additionally, definitional components of IIM-ILD will be extracted according to a pre-specified taxonomy comprising clinical, serologic, radiologic, functional, and multidisciplinary adjudication domains to support structured comparison across studies. Missing domains will be recorded as “not reported’’.

**Table 3.** List of data items to be extracted during the systematic literature review.

| **Item** | **Description** |
| --- | --- |
| Article and bibliographic information | Article title, bibliographic information and minimal descriptive data regarding the study including country, design, participating centers, sample and year of study |
| Demographics of study subjects | Female sex (frequency), age (central tendency measure) and ethnicity (frequency) |
| Description of study population (dichotomous, not exclusive) | Main type of patients included in the study, be it IIM population as a whole; specific IIM subtypes; juvenile IIM population; ILD population as a whole; ILD specific patterns; and/or patients with positive autoantibodies. |
| Myositis classification criteria applied (dichotomous, not exclusive) | Bohan & Peter’s (24, 25), ACR/EULAR 2017(26), ENMC criteria for ASyS (27), IMNM(28) and DM (29), Sontheimer’s criteria for CADM (30), Solomon’s (31) and Connors’ criteria for ASyS (32), CLASS criteria of ASyS (33) |
| IIM clinical features (frequency) | Clinical muscle weakness, arthritis, unexplained fever, Raynaud’s phenomenon, constitutional symptoms, DM cutaneous manifestations, ASyS cutaneous manifestations, pulmonary hypertension. |
| IIM Diagnostic Methods (dichotomous, not exclusive) | Clinical signs, autoantibodies, muscle enzymes elevation, MRI, EMG, muscle biopsy. |
| ILD classification criteria applied (dichotomous, not exclusive) | ATS/ERS 2013 (34) or other ILD criteria |
| ILD Diagnostic Methods (dichotomous, not exclusive) | CT scan, PFTs, bronchoalveolar lavage, lung biopsy |
| IIM subtype (frequency) | DM, CADM, IMNM, ASyS, polymyositis, juvenile DM, and other juvenile IIMs |
| ILD pattern (frequency) | UIP, NSIP, OP, NSIP+OP and other ILD patterns |
| IPAF (frequency) | Patients meeting the ATS/ERS IPAF criteria (10) |
| Average PFT data (% of predicted) | FVC and DLCO |
| Seropositivity rates (frequency) | MSA/MAA, other autoantibodies |
| Formal IIM-ILD definition | If a formal definition of IIM-ILD has been proposed in the study, it will be recorded |

*IIM: Idiopathic Inflammatory Myositis; ILD: Interstitial Lung Disease; ACR: American College of Rheumatology; EULAR: European Alliance of Associations for Rheumatology; ENMC: European Neuromuscular Centre; ASyS: Anti Synthetase Syndrome; IMNM: Immune-Mediated Necrotizing Myositis; DM: Dermatomyositis; CADM: Clinically Amyopathic Dermatomyositis; CLASS: The Classification Criteria for Antisynthetase Syndrome; MRI: Magnetic Resonance Imaging; EMG: Electromyogram; ATS: American Thoracic Society; ERS: European Respiratory Society; CT: computed tomography; PFT: pulmonary function test; UIP: Usual Interstitial Pneumonia; NSIP: Non-Specific Interstitial Pneumonia; OP: Organizing Pneumonia; IPAF: Interstitial Pneumonia with Autoimmune Features; FVC: Forced Vital Capacity; DLCO: Diffusing Capacity of Carbon Monoxide; MSA: Myositis-Specific Autoantibodies; MAA: Myositis-Associated Autoantibodies*

*Outcomes and Prioritization*

The primary outcome is a comprehensive overview and analysis of IIM-ILD definitions in the searched literature. This systematic literature review process will provide the groundwork for the Delphi process-based expert consensus to establish a uniform definition and nomenclature for IIM-ILD.

*Assessing Risk of Bias in Individual Studies*

Two reviewers will independently assess the risk of bias of each study, using the Cochrane Risk of Bias (RoB) 2 instrument (35) to assess RCTs and a validated tool to assess risk of bias for non-randomized studies of interventions, such as the Newcastle-Ottawa tool (36). In this definition-focused review, risk-of-bias assessment is intended to characterize the methodological context in which definitions of IIM-ILD are generated, rather than to rank or recommend specific definitions.

Accordingly, findings will be interpreted in light of study quality, with results stratified by study design and risk-of-bias categories where feasible. Studies at higher risk of bias will not be excluded; however, their definitions will be explicitly flagged and interpreted with appropriate caution. Risk-of-bias assessment will also be used to explore whether heterogeneity in definitions reflects underlying methodological limitations.

Discrepancies between reviewers will be resolved by an independent adjudicator. Consistent with the aim of this review to map rather than endorse definitions, risk-of-bias evaluation will inform interpretation but will not determine study inclusion or weighting in the synthesis. The results of all bias assessments will be reported in the final manuscript as a supplement.

*Data Synthesis*

Data studies will be analyzed if they meet all inclusion and exclusion criteria outlined in the protocol, report sufficient data relevant to the definitions, classifications, or diagnostic criteria for IIM-ILD, and contain independent data from other included studies. Studies on the pediatric population will be analyzed separately from the adult population.

This review aims at characterizing and harmonizing how IIM-ILD is defined in the literature, rather than as a traditional outcome-focused meta-analysis. Thus, the primary unit of analysis will be the IIM-ILD definition, rather the study as a whole and definitional components will serve as secondary units of comparison across definitions. A structured narrative synthesis will be performed. This will include a thematic analysis of definitions and classification criteria used across studies; tabulated comparisons of diagnostic criteria (e.g., antibody status, HRCT findings, muscle involvement); identification of key areas of agreement and variation in IIM-ILD definitions; and synthesis of facilitators and challenges in applying each definition in research and clinical settings. The strength and consistency of evidence will be assessed, and recommendations for future research, including minimum reporting criteria and candidate elements for a unified definition, will be proposed. When these criteria are met, frequencies and proportions describing the use of specific IIM-ILD definitions will be reported in all studies. Definitions will also be interpreted according to their context of use. Specifically, we will distinguish whether definitions are applied for trial eligibility, epidemiologic or observational cohort classification, clinical diagnosis, guideline or consensus recommendations, or other research purposes. Study design characteristics, including randomized trials, observational cohorts, case-control studies, guidelines, and consensus statements, will be used to support this categorization. This contextual stratification will allow us to examine whether variation in IIM-ILD definitions reflects differences in intended use, rather than treating all definitions as directly equivalent.

Although surgical lung biopsy is more commonly reported in earlier literature, contemporary evaluation of ILD in SARD - including IIM - prioritizes HRCT as the central diagnostic modality, as reflected in the 2023 ACR/CHEST guideline for screening and monitoring of SARD-associated ILD (8). Accordingly, HRCT-defined ILD patterns will serve as the principal reference framework for synthesis in this review, with histopathology treated as supportive or contextual when reported. When biopsy findings conflict with HRCT patterns, the HRCT-based classification will be prioritized for comparative synthesis, and discordance will be documented narratively. Studies in which histology is used as a mandatory defining criterion will be categorized as employing a “histology-dependent definition” and, where feasible, analyzed separately to preserve interpretability across evolving diagnostic paradigms.

In order to ensure a standardized method to manage data derived by studies lacking explicit or consistent definitions of IIM-ILD, the SLR will be refined for handling missing or inconsistent definitional details. In particular, papers mentioning ILD but not providing a definition will be included to capture real-world use of the term IIM-ILD but they will be categorized as “no explicit definition provided: the definition of IIM-ILD only derives from inclusion and exclusion criteria in the study but is not formally stated”. These studies will be analyzed narratively. If partial components of ILD definition are provided (e.g. diagnosis based on HRCT without specifying patterns or "clinician diagnosis") studies will be included. All available components of the definition will be extracted and reported into the standardized data extraction form and missing elements will be recorded as “not reported”. Finally, if inconsistent, multiple or conflicting definitions are provided in different sections of a paper, each definition will be extracted and analyzed separately where feasible. An attempt to contact the Authors of the study will be managed, if feasible. The different definitions of ILD will be registered and the inconsistency will be documented.

A meta-analysis is not planned as a core objective, but may be undertaken in very limited circumstances if sufficiently homogeneous and comparable data exist. Specifically, quantitative pooling will only be considered when: (1) ≥4 studies evaluate the same predefined definitional component or outcome; (2) sufficient numerical data are available to permit pooling.

Studies with missing or insufficient data for particular outcomes or exposures of interest will be excluded. Where similar outcomes are reported using different scales or metrics, we will standardize or transform data to a common format where methodologically appropriate. The data will be synthesized based on classification frameworks/criteria used as well as IIM subtypes. A pre-specified and standardized taxonomy of definitional components will be used to enhance reproducibility, transparent documentation and interpretability of the final synthesis. This taxonomy will categorize definitional elements into five core domains: (1) Clinical (e.g., respiratory symptoms, temporal relationship of ILD to myositis onset); (2) Serologic (e.g., inclusion or exclusion of myositis-specific or myositis-associated autoantibodies); (3) Radiologic (e.g., HRCT-based ILD confirmation and specific pattern requirements such as UIP/NSIP/OP); (4) Functional (e.g., pulmonary function test thresholds such as FVC or DLCO cut-offs); and (5) Multidisciplinary adjudication (e.g., requirement for multidisciplinary discussion or consensus diagnosis). Missing domains will be explicitly recorded as “not reported.” Formal prioritization of candidate core elements will not be undertaken within this review, but will be deferred to the planned Delphi process.

The provenance of each IIM-ILD definition will be recorded where reported, including whether it was newly proposed, adapted from prior guidelines or consensus statements, derived from existing criteria, or inferred from study eligibility criteria. This will allow definitions to be interpreted according to their source and context of use, while minimizing overrepresentation due to repeated citation or reuse. Where feasible, we will emphasize independent application across studies rather than citation frequency alone. Potential overlapping populations will be assessed through a structured comparison of the following study characteristics: recruiting institution(s) and geographic location, study period (years of patient enrollment), sample size and detailed inclusion/exclusion criteria, author lists and classification criteria employed for disease definitions. In case of overlapping cohorts, the study with larger sample size, most comprehensive dataset or highest-quality data (e.g., more detailed methodology) will be prioritized. Where study quality is equivalent, preference will be given based on the scope of IIM-ILD definitions used, prioritizing those that incorporate both MSA and myositis-associated antibodies (MAA).  All extracted data will be harmonized to ensure comparability across studies. When feasible, a meta-analysis will be conducted using a random-effects model to account for expected variability across study designs, patient populations, and IIM-ILD definitions. We will assess between-study variability using the I² statistic, and Cochrane’s Q test will be used to evaluate whether statistically significant differences are present across studies. Subgroup analyses (e.g., by inclusion of MAA/MSA and the presence of muscle symptoms) and meta-regression (where appropriate) will be conducted to explore sources of heterogeneity. Results will be visualized with forest plots and, if applicable, funnel plots to assess publication bias. If a sufficient number of studies are available, subgroup analyses will be conducted in pre-specified domains: antibody status (MSA-positive, MAA-positive, or seronegative), study design (cohort versus case-control), geographic region, and risk of bias. Hypotheses include potential differences in outcomes between antibody-defined subgroups, the influence of study design on reported prevalence and outcomes, regional variations reflecting differences in clinical practice or testing availability, and the sensitivity of results to the exclusion of high risk of bias studies (e.g., non-randomized design, small sample size, unclear ILD definition). To assess the certainty of the body of evidence, we will apply the Grading of Recommendations Assessment, Development and Evaluation (GRADE) approach as recommended by Cochrane (37-39).

**Discussion**

The protocol outlines the first literature review to systematically identify, evaluate, and synthesize published definitions, classifications, and diagnostic criteria for IIM-ILD. By mapping the spectrum of definitions used across various study designs and clinical contexts, this work seeks not only to identify areas of consensus, but also to highlight inconsistencies. Current variability in IIM-ILD definitions represent a significant barrier due to inconsistent patient phenotyping, and reduced comparability across studies. The findings will directly aid in the development of guidelines and future expert Delphi procedures. The final goal is dual. First, to improve clinical trial readiness with the development of a standardized definition and unified nomenclature, Second, to support the integration of pulmonary involvement into future IIM classification criteria.

Specifically, definitional components identified in the literature - such as clinical features, HRCT findings, autoantibody profiles, and diagnostic thresholds - will be compiled as potential elements of an IIM-ILD definition to be evaluated by the Delphi expert panel. Features that are consistently described will be proposed as core candidate items, whereas components that are less frequently reported, inconsistently defined, or emerging in recent literature will be specifically highlighted for expert discussion during the Delphi process. This approach ensures that the Delphi exercise is grounded in the available evidence while also allowing expert consensus to address areas where the literature remains limited or heterogeneous. Publication of formal methodology for this project will form a basis for future IIM-ILD WG projects. The current protocol will serve as a guidance document for similar systematic literature review projects within the IIM-ILD WG and MCTC.

Several additional strengths of this protocol should be emphasized. We have a dedicated interdisciplinary group working under MCTC which includes senior steering committee oversight. The project will apply a rigorous methodology, with clearly defined team roles and responsibilities. Furthermore, the project leaders and contributors bring substantial expertise and a strong publication record in IIM research, reinforcing the credibility and impact of the work.

Several limitations are also anticipated. First, we expect substantial variability in study designs and in diagnostic and classification criteria across studies, including variations in methodology, patient selection, and clinical assessment, which may complicate the comparability of findings. In this setting, potential for publication bias may arise from more severe or clinically overt disease phenotypes, as such cases are more likely to be captured in specialized centers and reported in the literature. This may limit the representativeness of milder or subclinical ILD presentations. Second, diagnosis of ILD was based on HRCT. Restricting ILD definitions to HRCT-based criteria may introduce selection bias and potentially exclude some studies, particularly earlier reports or those relying on histopathology or pulmonary function tests. Our decision to prioritize HRCT-based definitions was grounded in contemporary clinical practice, where HRCT represents the cornerstone of ILD diagnosis and is used in the vast majority of cases. This is particularly relevant in connective tissue disease–associated ILD, where surgical lung biopsy is rarely performed. We did not include PFT-based definitions, as pulmonary function abnormalities in myositis can be due to several reasons and may lack specificity for ILD, potentially reducing diagnostic precision. Moreover, differences in the inclusion or exclusion of specific MSA and MAA should be considered. This definitional variability may be further compounded by differences in antibody assay techniques and platforms across studies, which could affect antibody classification and comparability. Third, geographic variability in diagnostic practices, including differences in access to HRCT, autoantibody testing and multidisciplinary evaluation may contribute to heterogeneity in the published literature and introduce publication bias. Indeed, certain geographic areas, particularly East Asia, have a disproportionately high volume of ILD-related research. This imbalance may influence the representation of disease phenotypes, diagnostic approaches, and clinical perspectives in the available literature. In addition, we acknowledge that exclusion of non-English and grey literature may introduce risk of geographic bias and regional data underrepresentation, limiting the generalizability of findings across diverse geographic and clinical contexts. However, the review will focus exclusively on peer-reviewed evidence, thus ensuring methodological rigor and excluding emerging nomenclature or localized diagnostic frameworks that have not yet reached formal publication. Fourth, variability in radiologic expertise and interpretation standards across centers and studies may affect the consistency of imaging-based ILD classification, particularly in the absence of centralized image review or standardized reporting criteria.

Fifth, while subgroup analyses are planned to explore differences by antibody status, geographic region, and other variables, the number of eligible studies for certain subgroups may be small, potentially reducing statistical power and limiting the generalizability of findings. In addition, the inclusion of consensus statements and clinical guidelines - while valuable for understanding prevailing expert opinion - may introduce bias, as such documents do not always rely on empirical data and may reflect expert agreement rather than systematically generated evidence.

Finally, despite the global authorship and intended international relevance of this work, important considerations related to equity and global applicability must be acknowledged. Access to key diagnostic resources for ILD - such as HRCT, extended MSA testing and formal multidisciplinary discussion - varies substantially across regions and healthcare systems worldwide, particularly between high- and low-resource settings. Future consensus definitions should therefore balance scientific rigor with feasibility and include options adaptable to resource-variable settings. This may improve global uptake and real-world implementation across diverse practice environments. Consequently, many existing definitions of IIM-ILD have been developed primarily in specialized centers with advanced diagnostic infrastructure, which may limit their applicability in settings where such resources are not routinely available. Professional society statements emphasize the importance of multidisciplinary evaluation in ILD diagnosis and acknowledge that this approach is not universally feasible due to variability in access to imaging and serologic testing across regions. Recently, consensus-based recommendations for improving equity in ILD research and enhancing the generalizability and applicability of findings to the global ILD population have been developed (40).

These limitations will be addressed through transparent documentation of sources of heterogeneity, careful appraisal of study methodology, and the use of robust narrative synthesis approaches when quantitative pooling is not feasible. Subgroup analyses will be interpreted cautiously, particularly where data are sparse. Even with these challenges, bringing together and critically examining the available literature should lay a strong foundation for building a consensus definition and standardized terminology for IIM-ILD. Moreover, these findings are expected to inform subsequent consensus-based efforts that explicitly consider feasibility, equity, and global applicability, in order to ensure that future IIM-ILD definitions are both scientifically robust and broadly implementable across diverse healthcare environments.

An additional consideration relates to the inclusion of pediatric populations with juvenile idiopathic inflammatory myopathies (JIIM). Disease expression in juvenile forms of myositis may differ from adult disease, with pulmonary involvement sometimes being subclinical. Furthermore, diagnostic evaluation in pediatric populations may be limited by the lack of standard assessments commonly used in adult ILD studies. Pediatric cohorts are also typically smaller and less extensively characterized, particularly with respect to serologic testing and imaging-based phenotyping. As a result, extrapolation of adult-derived definitions and diagnostic thresholds for IIM-ILD to pediatric populations should be approached cautiously. By explicitly including juvenile studies and analyzing them separately from adult populations, this review aims to identify these gaps and highlight the need for pediatric-informed definitional frameworks.

In summary, our protocol and systematic literature review is the first concrete step towards standardizing nomenclature for adults and juveniles with IIM-ILD. Understanding the spectrum of IIM-ILD definitions used in the literature will aid in creating the uniform definition of IIM-ILD. The outcomes of this protocol will serve as an evidence-informed roadmap for Delphi-based consensus development. Other anticipated downstream outputs could include a minimum reporting set for future IIM-ILD studies, guide variable list for registries, clinical trials and future methodological studies.

**List of abbreviations**

ACR – American College of Rheumatology

ASyS – Antisynthetase Syndrome

ATS – American Thoracic Society

CADM – Clinically Amyopathic Dermatomyositis

CHEST – American College of Chest Physicians

DLCO – Diffusing Capacity of Carbon Monoxide

DM – Dermatomyositis

EMG – Electromyogram

ENMC – European Neuromuscular Centre

ERS – European Respiratory Society

EULAR – European Alliance of Associations for Rheumatology

FVC – Forced Vital Capacity

GRADE – Grading of Recommendations Assessment, Development and Evaluation

HRCT – High Resolution Computer Tomography

IIM – Idiopathic Inflammatory Myopathies

IIM-ILD – Interstitial Lung Disease Related to Idiopathic Inflammatory Myositis

ILD – Interstitial Lung Disease

IMNM – Immune-Mediated Necrotizing Myositis

IPAF – Interstitial Pneumonia with Autoimmune Features

MAA – Myositis-Associated Antibodies

MCTC – Myositis Clinical Trials Consortium

MeSH – Medical Subject Headings for inflammatory muscle diseases

MRI – Magnetic Resonance Imaging

MSA – Myositis-Specific Antibodies

NSIP – Non-Specific Interstitial Pneumonia

OP – Organizing Pneumonia

PFT – Pulmonary Function Tests

PICO – Population, Intervention, Comparison, Outcome

PRESS – Peer Review of Electronic Search Strategies

PRISMA – Preferred Reporting Items for Systematic Reviews and Meta-Analyses

RCTs – Randomized Controlled Trials

RoB – Cochrane Risk of Bias

SARDs – Systemic Autoimmune Rheumatic Diseases

SHARE – Single Hub and Access point for paediatric Rheumatology in Europe

SLR – Systematic Literature Review

UIP – Usual Interstitial Pneumonia

WG – Working Group

**Declarations**

**Ethics approval and consent to participate**

Not applicable

**Consent for publication**

Not applicable

**Availability of data and materials**

Not applicable

**Competing interests**

The authors declare no competing interests.

**Funding/Sponsoring**

No funding has been received for this work.

**Authors’ contributions**

Konstantinos Triantafyllias; Investigation, conceptualization, validation, project administration, writing, reviewing, editing of final protocol, review guarantor.

Ramiro Gomez: Investigation, conceptualization, data curation, validation, project administration, reviewing and editing of the final protocol.

Jisna Paul: Investigation, conceptualization, supervision, project administration, writing, reviewing, and editing of final protocol.

Simone Appenzeller: Investigation, conceptualization, validation, resources, reviewing and editing of the final protocol.

Elena Bartoloni: Investigation, conceptualization, validation, reviewing and editing of the final protocol.

Daniel Brito de Araujo: Investigation, conceptualization, validation, resources, reviewing and editing of the final protocol.

Solciris A Dominguez: Investigation, conceptualization, resources, reviewing and editing of the final protocol.

Komal Ejaz: Investigation, conceptualization, PROSPERO registration, reviewing and editing of final protocol.

Antonis Fanouriakis: Investigation, conceptualization, reviewing and editing of the final protocol.

Kristin Houghton: Investigation, conceptualization, reviewing and editing of the final protocol.

Marc Janssen: Investigation, conceptualization, reviewing and editing of the final protocol.

Chandana Keshavamurthy: Investigation, conceptualization, validation, writing, reviewing, and editing of the final protocol.

Marina Khoury: Methodology, data curation, resources, reviewing and editing of the final protocol.

Shaye Kivity: Investigation, conceptualization, validation, reviewing, and editing of the final protocol.

Océane Landon-Cardinal: Investigation, conceptualization, reviewing and editing of the final protocol.

Ignacio Lopez: Investigation, conceptualization, validation, data curation, reviewing and editing of the final protocol.

Nikolaos Marketos: Investigator, conceptualization, validation, reviewing, and editing of the final protocol.

Edoardo Marrani: Investigation, conceptualization, resources, reviewing and editing of the final protocol.

Jan Willem Marsden: Investigation, conceptualization, reviewing and editing of the final protocol.

Clio Mavragani: Investigation, conceptualization, validation, reviewing and editing of the final protocol.

Pintip	Ngamjanyaporn: Investigation, conceptualization, validation, reviewing and editing of the final protocol.

Apostolos Perelas: Investigation, conceptualization, reviewing and editing of the final protocol.

Victor Pimentel-Quiroz: Investigation, conceptualization, validation, reviewing and editing of the final protocol.

Vasilios Tzilas: Investigation, conceptualization, reviewing and editing of the final protocol.

Juan David Urriago Gil: Investigation, validation, data curation, reviewing and editing of final protocol.

Susan Vehar: Investigation, conceptualization, reviewing and editing of the final protocol

Gwen Wilson: Methodology, validation, resources, reviewing and editing of the final protocol

Erin Wilfong: Investigation, conceptualization, supervision, project administration, reviewing and editing of the final protocol

Charalampia Papadopoulou: Investigation, conceptualization, supervision, project administration, reviewing and editing of the final protocol.

Rohit Aggarwal: Investigation, conceptualization, supervision, project administration, reviewing and editing of the final protocol.

Robert Hallowell: Investigation, conceptualization, supervision, project administration, reviewing and editing of the final protocol.

Vivek Nagaraja: Investigation, conceptualization, supervision, project administration, reviewing and editing of the final protocol.

Faye Pais: Investigation, conceptualization, supervision, project administration, reviewing and editing of the final protocol

Elena K. Joerns: Investigation, conceptualization, validation, resources, supervision, project administration, writing, reviewing, editing of final protocol, review guarantor.

**Acknowledgements**

n/a

**Authors’ information**

[ktriantafyllias@gmail.com](mailto:ktriantafyllias@gmail.com), ramiroadriangomez@gmail.com, Jisna.Paul@osumc.edu, appenzellersimone@gmail.com, [elena.bartolonibocci@unipg.it](mailto:elena.bartolonibocci@unipg.it), alejandro.benitez@institutocer.com, araujodb@gmail.com, SAD408@pitt.edu, Komal.Ejaz@piedmont.org,  afanour@med.uoa.gr, [khoughton@cw.bc.ca](mailto:khoughton@cw.bc.ca), m.h.a.jansen@umcutrecht.nl, murtychandana@gmail.com, marina.khoury@gmail.com, kivitys@gmail.com, [oceane.landon-cardinal@umontreal.ca](mailto:oceane.landon-cardinal@umontreal.ca), ignacio.lopez.1989@gmail.com, [nickmarketos@yahoo.com](mailto:nickmarketos@yahoo.com), [edoardo.marrani@meyer.it](mailto:edoardo.marrani@meyer.it), J.W.N.Marsden@umcutrecht.nl, cliopmavragani@gmail.com,  [pintip.nga@mahidol.ac.th](mailto:pintip.nga@mahidol.ac.th), Apostolos.Perelas@vcuhealth.org, victorpq4@gmail.com, tzilasvasilios@gmail.com, j.urreuma23@gmail.com, [svehar@miami.edu](mailto:svehar@miami.edu), Wilson.Gwen@mayo.edu, erin.m.wilfong@vumc.org, charalampia.papadopoulou@gosh.nhs.uk, aggarwalr@upmc.edu, rhallowell@mgh.harvard.edu, Nagaraja.Vivek@mayo.edu, Faye.Pais@medicine.ufl.edu, [Joerns.Elena@mayo.edu](mailto:Joerns.Elena@mayo.edu).

**ORCID ID’s**

Konstantinos Triantafyllias: 0000-0001-8764-2636

Ramiro Gomez: 0000-0003-2991-1070

Simone Appenzeller: 0000-0001-5075-4474

Elena Bartoloni: [0000-0003-4776-2136](https://orcid.org/0000-0003-4776-2136)

Alejandro Benitez: 0009-0001-3222-8574

Daniel Brito de Araujo: 0000-0002-4840-945X

Komal Ejaz: 0000-0001-9920-289X

Antonis Fanouriakis: 0000-0003-2696-031X

Kristin Houghton: 0000-0001-7174-1007

Chandana Keshavamurthy: [0000-0001-7171-9379](https://orcid.org/0000-0001-7171-9379?lang=en)

Marina Khoury: 0000-0002-8696-6871

Shaye Kivity: 0000-0002-8391-5446

Océane Landon-Cardina: 0000-0002-3361-6165

Ignacio Lopez: 0009-0006-3403-6732

Nikolaos Marketos: 0000-0001-6995-3713

Edoardo Marrani: 0000-0002-0346-3268

Jan Willem Marsden: 0009-0008-8503-4969

Clio Mavragani: 0000-0001-6291-6290

Pintip Ngamjanyaporn: [0000-0001-7948-1748](https://orcid.org/0000-0001-7948-1748)

Victor Pimentel-Quiroz: 0000-0002-3638-7054

Vasilios Tzilas: [0000-0002-7030-6166](https://orcid.org/0000-0002-7030-6166)

Juan David Urriago Gil: 0009-0001-3180-6184

Susan Vehar: 0000-0003-4136-8488

Gwen Wilson: [0000-0002-7214-0403](https://orcid.org/0000-0002-7214-0403)

Erin Wilfong: 0000-0002-6584-1578

Charalampia Papadopoulou: [0000-0002-1237-0557](https://orcid.org/0000-0002-1237-0557)

Rohit Aggarwal: [0000-0001-7531-8038](https://orcid.org/0000-0001-7531-8038)

Robert Hallowell: 0000-0001-9386-7700

Vivek Nagaraja: 0000-0002-4930-3200

Faye Pais: 0000-0003-4058-5309

Elena K. Joerns: 0000-0001-7089-9152

[ktriantafyllias@gmail.com](mailto:ktriantafyllias@gmail.com), ramiroadriangomez@gmail.com, Jisna.Paul@osumc.edu, appenzellersimone@gmail.com, [elena.bartolonibocci@unipg.it](mailto:elena.bartolonibocci@unipg.it), alejandro.benitez@institutocer.com, araujodb@gmail.com, SAD408@pitt.edu, [ejazk@ccf.org](mailto:ejazk@ccf.org),  afanour@med.uoa.gr, [khoughton@cw.bc.ca](mailto:khoughton@cw.bc.ca), m.h.a.jansen@umcutrecht.nl, chandana.keshavamurthy@ochsner.org, marina.khoury@gmail.com, kivitys@gmail.com, [oceane.landon-cardinal@umontreal.ca](mailto:oceane.landon-cardinal@umontreal.ca), ignacio.lopez.1989@gmail.com, [nickmarketos@yahoo.com](mailto:nickmarketos@yahoo.com), [edoardo.marrani@meyer.it](mailto:edoardo.marrani@meyer.it), J.W.N.Marsden@umcutrecht.nl, [mauragan@med.uoa.gr](mailto:mauragan@med.uoa.gr),  [pintip.nga@mahidol.ac.th](mailto:pintip.nga@mahidol.ac.th), Apostolos.Perelas@vcuhealth.org, victorpq4@gmail.com, tzilasvasilios@gmail.com, j.urreuma23@gmail.com, [svehar@miami.edu](mailto:svehar@miami.edu), Wilson.Gwen@mayo.edu, erin.m.wilfong@vumc.org, charalampia.papadopoulou@gosh.nhs.uk, aggarwalr@upmc.edu, rhallowell@mgh.harvard.edu, Nagaraja.Vivek@mayo.edu, Faye.Pais@medicine.ufl.edu, [Joerns.Elena@mayo.edu](mailto:Joerns.Elena@mayo.edu).

**Protocol Amendments** If we need to amend this protocol, we will list in this section the date of each amendment, describe the change made, and provide the rationale for making it. Each amendment will also be documented by modifying the appropriate section(s) in the protocol and annotating the modification with the date on which the modification was made.

**Additional notes**

We will include the exact bibliographic database search strategy as well as the data used in the review as supplements in our manuscript publication.

**Additional files**

Supplementary file 1: PRISMA-P 2015 Checklist

Supplementary file 2: Glossary of Key Terms

**References**

1.	Mariampillai K, Granger B, Amelin D, Guiguet M, Hachulla E, Maurier F, et al. Development of a New Classification System for Idiopathic Inflammatory Myopathies Based on Clinical Manifestations and Myositis-Specific Autoantibodies. JAMA Neurol. 2018;75(12):1528-37.

2. Barba T, Fort R, Cottin V, Provencher S, Durieu I, Jardel S, et al. Treatment of idiopathic inflammatory myositis associated interstitial lung disease: A systematic review and meta-analysis. Autoimmun Rev. 2019;18(2):113-22.

3. Kiely PD, Chua F**.** Interstitial lung disease in inflammatory myopathies: clinical phenotypes and prognosis. Curr Rheumatol Rep. 2013;15(9):359.

4.	Hallowell RW, Danoff SK**.** Diagnosis and Management of Myositis-Associated Lung Disease. Chest. 2023;163(6):1476-91.

5.	Zanframundo G, Dourado E, Bauer-Ventura I, Faghihi-Kashani S, Yoshida A, Loganathan A, et al. The role of multicriteria decision analysis in the development of candidate classification criteria for antisynthetase syndrome: analysis from the CLASS project. Ann Rheum Dis. 2025.

6.	Li S, Sun Y, Shao C, Huang H, Wang Q, Xu K, et al. Prognosis of adult idiopathic inflammatory myopathy-associated interstitial lung disease: a retrospective study of 679 adult cases. Rheumatology (Oxford). 2020.

7.	Bellutti Enders F, Bader-Meunier B, Baildam E, Constantin T, Dolezalova P, Feldman BM, et al. Consensus-based recommendations for the management of juvenile dermatomyositis. Ann Rheum Dis. 2017;76(2):329-40.

8.	Johnson SR, Bernstein EJ, Bolster MB, Chung JH, Danoff SK, George MD, et al. 2023 American College of Rheumatology (ACR)/American College of Chest Physicians (CHEST) Guideline for the Screening and Monitoring of Interstitial Lung Disease in People with Systemic Autoimmune Rheumatic Diseases. Arthritis Care Res (Hoboken). 2024;76(8):1070-82.

9.	Johnson SR, Bernstein EJ, Bolster MB, Chung JH, Danoff SK, George MD, et al. 2023 American College of Rheumatology (ACR)/American College of Chest Physicians (CHEST) Guideline for the Treatment of Interstitial Lung Disease in People with Systemic Autoimmune Rheumatic Diseases. Arthritis Care Res (Hoboken). 2024;76(8):1051-69.

10.	Fischer A, Antoniou KM, Brown KK, Cadranel J, Corte TJ, du Bois RM, et al. An official European Respiratory Society/American Thoracic Society research statement: interstitial pneumonia with autoimmune features. Eur Respir J. 2015;46(4):976-87.

11.	Graham J, Bauer Ventura I, Newton CA, Lee C, Boctor N, Pugashetti JV, et al. Myositis-specific Antibodies Identify A Distinct Interstitial Pneumonia with Autoimmune Features Phenotype. Eur Respir J. 2020.

12.	Bishnoi A, Tang IYK, Yoshida A, Pais FM, Usman SY, Dominguez SA, et al. The Myositis Clinical Trials Consortium: an international collaborative initiative to promote clinical trials in adult and juvenile myositis. Clin Exp Rheumatol. 2025;43(2):202-10.

13.	Moher D, Shamseer L, Clarke M, Ghersi D, Liberati A, Petticrew M, Shekelle P, Stewart LA; PRISMA-P Group. Preferred reporting items for systematic review and meta-analysis protocols (PRISMA-P) 2015 statement. *Syst Rev*. 2015;4(1):1. doi: 10.1186/2046-4053-4-1.

14	Page MJ, McKenzie JE, Bossuyt PM, Boutron I, Hoffmann TC, Mulrow CD, Shamseer L, Tetzlaff JM, Akl EA, Brennan SE, Chou R, Glanville J, Grimshaw JM, Hróbjartsson A, Lalu MM, Li T, Loder EW, Mayo-Wilson E, McDonald S, McGuinness LA, Stewart LA, Thomas J, Tricco AC, Welch VA, Whiting P, Moher D. The PRISMA 2020 statement: an updated guideline for reporting systematic reviews. BMJ. 2021 Mar 29;372:n71.

15.	Correia BP, Campanilho-Marques R, Dourado E, Silva M, Silva A, Costa F, et al. Myositis-Associated Interstitial Lung Disease: The Experience of a Tertiary Center. J Clin Med. 2024;13(20).

16.	Tian J, Ha YJ, Lee JS, Lee EY, Goldin JG, Kim GJ**.** Application of a Growth-Rate Model to Enhance Subgroup Identification in Heterogeneous Clinical Courses of the Idiopathic Inflammatory Myopathy-Associated Interstitial Lung Disease and Its Prognostic Implication. Int J Rheum Dis. 2025;28(6):e70320.

17.	Zhang W, Huang G, Zheng S, Lin J, Hu S, Zhuang J, et al. Risk prediction modelling in idiopathic inflammatory myositis-associated interstitial lung disease based on seven factors including serum KL-6 and lung ultrasound B-lines. Clin Exp Rheumatol. 2025;43(2):260-8.

18.	Wang H, Wang Y, Sun D, Yu S, Du X, Ye Q**.** Progressive pulmonary fibrosis in myositis-specific antibody-positive interstitial pneumonia: a retrospective cohort study. Front Med (Lausanne). 2023;10:1325082.

19.	Selva-O'Callaghan A, Labrador-Horrillo M, Muñoz-Gall X, Martínez-Gomez X, Majó-Masferrer J, Solans-Laque R, et al. Polymyositis/dermatomyositis-associated lung disease: analysis of a series of 81 patients. Lupus. 2005;14(7):534-42.

20.	Aggarwal R, McBurney C, Schneider F, Yousem SA, Gibson KF, Lindell K, et al. Myositis-associated usual interstitial pneumonia has a better survival than idiopathic pulmonary fibrosis. Rheumatology (Oxford). 2017;56(3):384-9.

21.	Hannah JR, Lawrence A, Martinovic J, Naqvi M, Chua F, Kouranos V, et al. Antibody predictors of mortality and lung function trends in myositis spectrum interstitial lung disease. Rheumatology (Oxford). 2024;63(11):3080-90.

22.	McGowan J, Sampson M, Salzwedel DM, Cogo E, Foerster V, Lefebvre C**.** PRESS Peer Review of Electronic Search Strategies: 2015 Guideline Statement. J Clin Epidemiol. 2016;75:40-6.

23.	Covidence systematic review software. Melbourne, Australia: Veritas Health Innovation.

24.	Bohan A, Peter JB**.** Polymyositis and dermatomyositis (first of two parts). N Engl J Med. 1975;292(7):344-7.

25.	Bohan A, Peter JB**.** Polymyositis and dermatomyositis (second of two parts). N Engl J Med. 1975;292(8):403-7.

26.	Lundberg IE, Tjärnlund A, Bottai M, Werth VP, Pilkington C, Visser Md, et al. 2017 European League Against Rheumatism/American College of Rheumatology classification criteria for adult and juvenile idiopathic inflammatory myopathies and their major subgroups. Annals of the Rheumatic Diseases. 2017;76(12):1955-64.

27.	Stenzel W, Mammen AL, Gallay L, Holzer MT, Kleefeld F, Benveniste O, et al. 273rd ENMC International workshop: Clinico-Sero-morphological classification of the Antisynthetase syndrome. Amsterdam, The Netherlands, 27-29 October 2023. Neuromuscul Disord. 2024;45:104453.

28.	Allenbach Y, Mammen AL, Benveniste O, Stenzel W**.** 224th ENMC International Workshop:: Clinico-sero-pathological classification of immune-mediated necrotizing myopathies Zandvoort, The Netherlands, 14-16 October 2016. Neuromuscul Disord. 2018;28(1):87-99.

29.	Mammen AL, Allenbach Y, Stenzel W, Benveniste O**.** 239th ENMC International Workshop: Classification of dermatomyositis, Amsterdam, the Netherlands, 14-16 December 2018. Neuromuscul Disord. 2020;30(1):70-92.

30.	Gerami P, Schope JM, McDonald L, Walling HW, Sontheimer RD**.** A systematic review of adult-onset clinically amyopathic dermatomyositis (dermatomyositis siné myositis): a missing link within the spectrum of the idiopathic inflammatory myopathies. J Am Acad Dermatol. 2006;54(4):597-613.

31.	Solomon J, Swigris JJ, Brown KK**.** Myositis-related interstitial lung disease and antisynthetase syndrome. J Bras Pneumol. 2011;37(1):100-9.

32.	Connors GR, Christopher-Stine L, Oddis CV, Danoff SK**.** Interstitial lung disease associated with the idiopathic inflammatory myopathies: what progress has been made in the past 35 years? Chest. 2010;138(6):1464-74.

33.	Zanframundo G FKS, Yoshida A, Dourado E, Bauer Ventura I, Rivero Gallegos D, Loganathan A, Sambataro G, Bozan F, Bae S, Lim D, Cheli M, Yamano Y, Bonella F, Corte T, Doyle T, Fiorentino D, Gonzalez-Gay M, Hudson M, Kuwana M, Lundberg I, Mammen A, McHugh N, Miller F, Montecucco C, Oddis C, Rojas-Serrano J, Schmidt J, Scirè C, Selva-O'Callaghan A, Werth V, Rozza D, Morosini M, Bozzini S, Cavagna L, Aggarwal R. **.** The Classification Criteria for Anti-Synthetase Syndrome (Class) Project [abstract]. . Arthritis Rheumatol. .

34.	Travis WD, Costabel U, Hansell DM, King TE, Jr., Lynch DA, Nicholson AG, et al. An official American Thoracic Society/European Respiratory Society statement: Update of the international multidisciplinary classification of the idiopathic interstitial pneumonias. Am J Respir Crit Care Med. 2013;188(6):733-48.

35.	Sterne JAC, Savović J, Page MJ, Elbers RG, Blencowe NS, Boutron I, et al. RoB 2: a revised tool for assessing risk of bias in randomised trials. Bmj. 2019;366:l4898.

36.	Wells GA, Shea B, O’Connell D, Peterson J, Welch V, Losos M, et al. The Newcastle-Ottawa Scale (NOS) for assessing the quality of nonrandomised studies in meta-analyses. 2000.

37.	Brozek JL, Akl EA, Alonso-Coello P, Lang D, Jaeschke R, Williams JW, et al. Grading quality of evidence and strength of recommendations in clinical practice guidelines. Part 1 of 3. An overview of the GRADE approach and grading quality of evidence about interventions. Allergy. 2009;64(5):669-77.

38.	Brożek JL, Akl EA, Compalati E, Kreis J, Terracciano L, Fiocchi A, et al. Grading quality of evidence and strength of recommendations in clinical practice guidelines part 3 of 3. The GRADE approach to developing recommendations. Allergy. 2011;66(5):588-95.

39.	Brozek JL, Akl EA, Jaeschke R, Lang DM, Bossuyt P, Glasziou P, et al. Grading quality of evidence and strength of recommendations in clinical practice guidelines: Part 2 of 3. The GRADE approach to grading quality of evidence about diagnostic tests and strategies. Allergy. 2009;64(8):1109-16.

40.	Assayag D, Adegunsoye A, Khor YH, Bonella F, Borie R, Buendia I, et al.. Racial, ethnic, sex, and gender equity for inclusive interstitial lung disease research: an official American Thoracic Society Research Statement. Am J Respir Crit Care Med. 2025;211(12):2251-2267.
