# Supplementary material for: Defining interstitial lung disease related to idiopathic inflammatory myopathies: a systematic review protocol of the Myositis Clinical Trial Consortium (MCTC)
Source: Syst Rev. 2026 Jun 26;15:252. doi: 10.1186/s13643-026-03230-w (PMC13573363; doi:10.1186/s13643-026-03230-w)
Supplement: Supplementary file 2 [file 13643_2026_3230_MOESM2_ESM.docx]

**Glossary of Key Terms**

**Idiopathic Inflammatory Myopathies (IIM):** Chronic, multisystem immune-mediated disorders characterized by inflammatory muscle weakness and variable involvement of the skin, lungs, joints, and heart.

**Polymyositis (PM):** An idiopathic inflammatory myopathy characterized by subacute, symmetric proximal muscle weakness, elevated muscle enzymes, and endomysial CD8⁺ T-cell–mediated muscle fiber injury; considered a diagnosis of exclusion after ruling out other myopathies.

**Dermatomyositis (DM):** An autoimmune inflammatory myopathy characterized by hallmark cutaneous findings (heliotrope rash, Gottron’s papules) and proximal muscle weakness, driven by complement-mediated microvascular injury leading to perifascicular atrophy.

**Antisynthetase Syndrome (AS):** A systemic autoimmune disorder defined by anti–aminoacyl–tRNA synthetase antibodies and characterized by interstitial lung disease, inflammatory myopathy, arthritis, mechanic’s hands, Raynaud phenomenon, and fever.

**Overlap Myositis (OM):** An autoimmune inflammatory myopathy occurring with features of connective tissue disease (e.g., Raynaud phenomenon, arthritis, sclerodactyly, interstitial lung disease), often associated with myositis-related autoantibodies (anti-Ku, anti-PM/Scl, anti-U1-RNP, anti-Ro) that shape the clinical phenotype.

**Inclusion Body Myositis (IBM):** A slowly progressive inflammatory myopathy, typically in patients over 50, characterized by asymmetric distal and proximal weakness (finger flexors, wrist flexors, quadriceps), combined CD8⁺ T-cell–mediated inflammation and degenerative protein aggregation (rimmed vacuoles, amyloid deposits), and poor response to immunosuppressive therapy.

**Immune-Mediated Necrotizing Myopathy (IMNM):** A subtype of inflammatory myopathy characterized by rapidly progressive proximal muscle weakness, markedly elevated creatine kinase, and muscle biopsy showing myofiber necrosis with minimal lymphocytic infiltration. It is classified by autoantibody status (anti-SRP, anti-HMGCR, or seronegative), driven by humoral autoimmunity and complement-mediated muscle injury, and often requires aggressive immunotherapy.

**Amyopathic Dermatomyositis (ADM):** A subset of dermatomyositis with characteristic skin findings (heliotrope rash, Gottron’s papules) but no clinical or laboratory evidence of muscle involvement for at least six months, often associated with anti-MDA5 antibodies and a high risk of rapidly progressive interstitial lung disease and malignancy.

**Interstitial Lung Disease ( ILD ) Patterns**

**Usual Interstitial Pneumonia (UIP):** A histopathologic and radiologic pattern of interstitial lung disease with heterogeneous subpleural and basal fibrosis, patchy fibroblast foci, and microscopic honeycombing, typical of idiopathic pulmonary fibrosis but also seen in connective tissue disease, hypersensitivity pneumonitis, and drug-induced lung injury.

**Nonspecific Interstitial Pneumonia (NSIP):** An ILD pattern with temporally and spatially uniform interstitial inflammation or fibrosis, bilateral ground-glass opacities with subpleural sparing on HRCT, and absence of honeycombing or fibroblast foci; commonly associated with connective tissue disease and generally more responsive to immunosuppression than UIP.

**Organizing Pneumonia (OP):** A reversible lung injury pattern with intraluminal plugs of connective tissue (Masson bodies) in alveoli and bronchioles, often presenting with subacute flu-like illness, peripheral or subpleural consolidations with air bronchograms or reversed halo sign on HRCT, typically responsive to corticosteroids, and classified as cryptogenic (COP) when idiopathic or secondary when linked to infection, drugs, radiation, or connective tissue disease.

**Lymphocytic Interstitial Pneumonia (LIP):** A rare benign pulmonary lymphoproliferative disorder with diffuse polyclonal lymphoid infiltration of alveolar septa and airways, presenting on HRCT with ground-glass opacities, centrilobular or subpleural nodules, and thin-walled cysts. Strongly associated with immune dysregulation (e.g., Sjögren syndrome, HIV, CVID) and variably responsive to corticosteroids; diagnosis requires demonstration of polyclonality to exclude lymphoma.

**Progressive Pulmonary Fibrosis (PPF):** A phenotype of fibrosing ILD marked by worsening respiratory symptoms, decline in lung function (≥5–10% FVC), or radiologic progression over one year despite standard therapy, occurring across various ILDs including SARD-ILD, and potentially responsive to antifibrotic treatment.

**Rapidly Progressive Interstitial Lung Disease (RP-ILD):** A severe form of ILD marked by rapid decline to hypoxic respiratory failure over days to weeks, most commonly associated with anti-MDA5 dermatomyositis and antisynthetase syndrome, distinguished from progressive pulmonary fibrosis by its acute course, and requiring urgent immunosuppressive therapy.

**Autoimmune ILD classifications**

**Interstitial Pneumonia with Autoimmune Features (IPAF):** A research classification for patients with idiopathic interstitial pneumonia who exhibit clinical, serologic, or morphologic signs of autoimmunity but do not meet criteria for a defined connective tissue disease. Diagnosis requires features from at least two of three domains—clinical (e.g., Raynaud’s, mechanic’s hands, inflammatory arthritis), serologic (e.g., ANA ≥1:320, specific autoantibodies), and morphologic (e.g., NSIP/OP pattern, lymphoplasmacytic infiltration)—with prognosis intermediate between IPF and CTD-ILD and potential responsiveness to immunosuppression.

**Systemic Autoimmune Rheumatic Disease–Associated Interstitial Lung Disease (SARD-ILD):** Interstitial lung disease occurring in the setting of systemic autoimmune rheumatic diseases (e.g., rheumatoid arthritis, systemic sclerosis, IIM, mixed connective tissue disease, Sjögren’s), characterized by lung inflammation and/or fibrosis with variable radiologic patterns (commonly NSIP, UIP, or OP), representing a major source of morbidity and mortality and typically managed with multidisciplinary care and immunosuppressive therapy.

**Myositis-Specific Antibodies (MSAs):** Autoantibodies predominantly found in idiopathic inflammatory myopathies that target intracellular proteins and define distinct clinical subgroups, phenotypes, extramuscular manifestations, and prognoses. Present in ~two-thirds of IIM patients, MSAs are classified by disease association:

- **Antisynthetase Syndrome:** anti–aminoacyl-tRNA synthetases (anti-Jo-1, PL-7, PL-12, OJ, EJ, Ha, KS, Zo) — linked to ILD, mechanic’s hands, arthritis, Raynaud phenomenon
- **Dermatomyositis:** anti-Mi-2 (classic DM, good prognosis), anti-MDA5 (amyopathic DM, RP-ILD), anti-TIF1γ (malignancy risk >40 years), anti-NXP2 (juvenile DM, malignancy), anti-SAE
- **Immune-Mediated Necrotizing Myopathy:** anti-SRP, anti-HMGCR (statin-associated)
- **Inclusion Body Myositis:** anti-cN1A (high specificity, moderate sensitivity)

**Myositis-Associated Antibodies (MAAs):** Autoantibodies found in patients with idiopathic inflammatory myopathies that are not specific to myositis and also occur in other connective tissue diseases, often linked to overlap syndromes and extramuscular manifestations. Common MAAs include:

- **Anti-PM/Scl (PM-Scl-75, PM-Scl-100):** Strongly associated with overlap myositis–systemic sclerosis, mechanic’s hands, ILD, and arthritis.
- **Anti-Ku:** Linked to overlap myositis and systemic sclerosis; may show immune-mediated necrotizing myopathy on biopsy.
- **Anti-U1RNP:** Associated with mixed connective tissue disease, arthritis (~60%), DM-like skin features (~60%), ILD (~45%), and—when coexisting with anti-Ro52—pericarditis (~40%) and glomerulonephritis (~25%).
- **Anti-U3RNP:** Seen in systemic sclerosis and overlap syndromes.
- **Anti-Ro52:** Often co-occurs with MSAs (esp. antisynthetase antibodies) and predicts more severe ILD; with anti-U1RNP, increases risk of pericarditis and glomerulonephritis.
- **Anti-RNA polymerase:** Rarely reported, associated with systemic sclerosis overlap features.
